# Supplementary figures and images for: Using unsupervised machine learning to quantify physical activity from accelerometry in a diverse and rapidly changing population
Source: PLOS Digit Health. 2023 Apr 5;2(4):e0000220. doi: 10.1371/journal.pdig.0000220 (PMC10075441; doi:10.1371/journal.pdig.0000220)

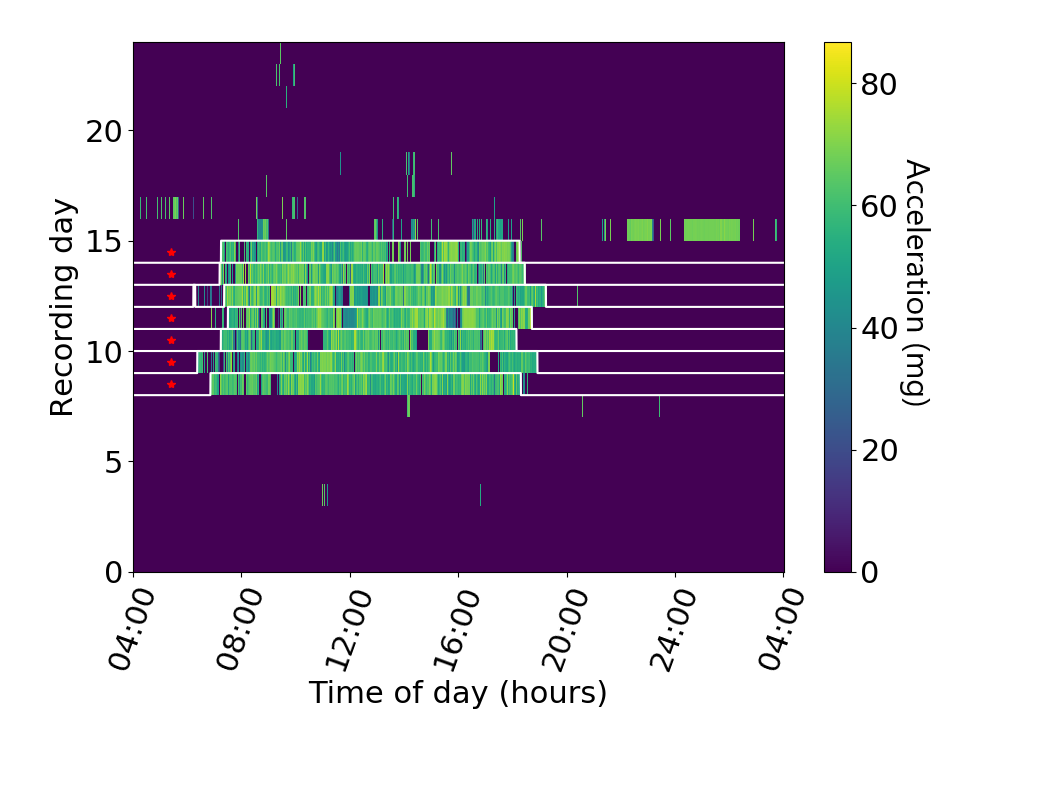

Supplement: S1 Fig — Red stars indicate the 7 days selected; the white line indicates wear time. (TIF) [file pdig.0000220.s001.tif]
