# Supplementary material for: Using unsupervised machine learning to quantify physical activity from accelerometry in a diverse and rapidly changing population
Source: PLOS Digit Health. 2023 Apr 5;2(4):e0000220. doi: 10.1371/journal.pdig.0000220 (PMC10075441; doi:10.1371/journal.pdig.0000220)
Supplement: Attachment — Submitted filename: rebuttal_letter.docx [file pdig.0000220.s002.docx]

Dear Editor,

We would like to thank the reviewers for their careful consideration of our paper and their very helpful comments and suggestions. We believe this has helped us to improve the clarity of the paper and develop further discussion of the results. Please see below our response to each of the reviewers’ comments.

Many thanks,

the authors.

| Reviewer Comment | Response + action |
| --- | --- |
| Reviewer #1: This paper investigates a Hidden Semi-Markov modelling approach for estimating activity levels in small children, going beyond traditional cut-point based approaches. The paper is relatively brief but is generally well written and presented with clear results and conclusions drawn. For improving the work I would suggest: | Thank you for reading our paper and for your kind comments, it is much appreciated. |
| (1) The authors state participants were recruited from "the universal healthy child pathway (i.e. health visitors)". I think this is a very UK focused phrasing and I would suggest internationalising the terminology used to make the work more accessible. Equivalent terms in other countries are given at https://ihv.org.uk/our-work/international/health-visiting-across-the-world/. | Response: Thank you. We have added from the Institute of Health Visitors, with reference to the suggested link, that “Different models of health visiting can be found across countries, under various labels (#REF )” Line 117. |
| (2) In the ENMO equation, the opening bracket is in the wrong place, it should be after the max. Moreover, why is the max operation needed? If the device is stationary it should be recording 1g and so this will evaluate to 0 anyway. If the device is moving, the acceleration should be >1g. Is this just to compensate for noise when the device is stationary which might lead to brief negative values? | The bracket has been moved to the correct location. Regarding the need for a max operation, you are right, we do this to ensure we don’t get negative values from noise. It is common practise to do this – for example in  Bakrania, K. et al. (2016) ‘Intensity thresholds on raw acceleration data: Euclidean norm minus one (ENMO) and mean amplitude deviation (MAD) approaches’, PLoS ONE, 11(10), p. e0164045. doi: 10.1371/journal.pone.0164045. [citation 20 in the paper]  We have updated the text to clarify this. Line 162. |
| (3) More information needs to be given around the chosen cut point values, in the text around line 182. It is stated many times that the "the best available thresholds for the population" are used, but with no discussion around this. For example, I see that the device used in [7] was worn at the waist, as in your study, but only by going and reading [7]. This information should be included here. In the discussion there should be a section on the thresholds used, and in what ways they are appropriate and in what ways they represent a limitation (despite being the best available). | Further information on the chosen cutpoints and how they were validated has been added to the methods section, lines 203-210.  Further discussion of the validity and the extent to which they are best available has been added to the discussion, lines 325 – 335. |
| (4) The caption for Fig. 2C needs improving - I struggled to see why the numbers didn't add up to 100% (until reaching this point in the text). Perhaps another colour for non-wear could be added. | Caption improved to describe non wear. Additional colour added to the key in the figure for non wear. Line 227 |
| Reviewer #2: The authors present a use case of the hidden Semi-Markov model (HSMM) with Bayesian inference to segment accelerometer data into physical behaviours in a paediatric population. They compare the activities segmented with HSMM with ones segmented using a cut point approach. They also compare the methods against domains of the PEDI-CAT, a report that evaluates disability in children.  Despite the use of HSMM not being novel, the authors provide evidence that the method can be used in a paediatric population with diverse motor abilities, evidence which is not currently available in the literature.  Overall, the paper is clear and well written. My main comment is that more details could be provided. More detailed comments and suggestions can be found below. | Thank you for reading our paper, and for your we have tried to provide additional details throughout the paper in the revisions we have made. |
| Abstract  • How do you define “best available” in the sentence at lines 30-31? | Updated to say: “thresholds from the literature which had been validated using the same device and for a population which most closely matched ours.” Lines 31 – 33. |
| • Consider adding how the measures listed in lines 32-33 were measured (especially for daily activity as to avoid confusion with the accelerometer-measured one). | We have clarified that the PEDI-CAT measures were taken by computer adaptive testing. Lines 30 – 31. |
| • Consider adding objective performance measures (e.g., correlation coefficient of unsupervised method VS one of cut-point approach). | Added comparisons of R2. Lines 34 - 36 |
| Introduction  • While you reference previous works using Hidden Semi-Markov Models (references 11 and 12), from the introduction it is not clearly stated that other studies have used HSMM for activity segmentation purposes. You have acknowledged that HSMM have been previously used for this purpose in the Discussion, but I would suggest you also mention this in the introduction and clearly state how your study is different. | This has now been added at lines 94 – 96. |
| • Reference 11 and 13 are the same. | Consolidated to reference 11. |
| Material and methods  • In the paragraph from lines 109-114 and in Table 1, the sample size is not consistent (278 VS 279). | The recruitment pathway was not available for one of the participants and so the proportion from recruitment pathway 1 was calculated out of 278. |
| • It would be useful to provide more information on the PEDI-CAT assessment, e.g., format (is it a questionnaire?), what variables takes in consideration and what is the output. Alternatively, please provide a reference to consult if the reader requires further information. | We have now provided a significant explanation of the PEDI-CAT here (lines 140 – 153) and pointed to further information which can be found in reference 19. |
| • At line 154-156 it is mentioned that parameter were selected using a sample of recordings – are these recordings from the same dataset analysed in the rest of the paper or is it different data? | These are recordings from this data set. Text updated to clarify this. Line 173 |
| • What is the rationale for modelling the durations with a Poisson distribution and the observations as a Gaussian distribution? Have you considered other distributions? | The rationale for using these distributions is that they have been successfully used previously for segmenting accelerometer data (reference 11). Future research could investigate other distributions but this would be outwith the scope of this current study. |
| • Line 194: it was not clear to me what you meant with “interruption”. | Updated to read: “This allowed 10% of an LPA bout or SED bout and 20% of an MVPA bout to be outside of the intensity range for that cut point (e.g. less than 28 counts per second for MVPA). By ignoring these interruptions, we are recognising that periods of activity may have short pauses.” Now lines 223 - 225 |
| • I suggest that you move Table 1 to the Results section. | We have placed table 1 in the methods because it is first mentioned in our data collection part of the methods. We feel it is necessary to reference the table when discussing the data collection and so have opted to keep the table here. |
| Results  • In Figure 2B, I suggest using a different colour palette for the cut-point approaches as it is hard to distinguish the segments on the graph. | Figure 2B has been updated to use a new colour palette for the cut-point approach. Line 227 |
| • Line 234: I suggest you don’t mention “agreement” as correlation cannot measure method agreement [1]. | Thank you for pointing this out and the reference, we have corrected this by removing mention of agreement. Now line 226. |
| • In order to clinically validate the methods by regressing the time spent in activities with PEDI-CAT domains, there must be evidence that physical behaviours can predict PEDI-CAT domains. If this evidence is available, please provide references. | The PEDI-CAT is the current-generation version of a well-established measure called the Pediatric Evaluation of Disability Inventory (PEDI), originally published in 1992 and since revised as a computer adaptive test (CAT). The PEDI-CAT measures children’s developmental abilities, as enacted in daily life, across daily activities, mobility and social-cognitive abilities, as well as the responsibility the child vs caregiver takes for managing complex, multi-step everyday life tasks. The PEDI-CAT has been validated for use across diagnostic groups, ages, settings and countries. Each of the PEDI-CAT domains has a response scale, and the PEDI-CAT software utilizes Item Response Theory statistical models to estimate a child’s abilities from a minimal number of the most relevant items or from a set number of items within each domain – ultimately establishing the child’s ability/difficulty and responsibility levels. Within the PEDI-CAT, the mobility domain assesses the child’s mobility behaviours, e.g. sitting, crawling, walking. It is one of the most long-established and widely used measures to assess children’s development, abilities, and disability across child health research and practice. We have added substantial description of the PEDI-CAT in the paper (lines 140 – 153), including a description of why we consider it to provide a clinically credible marker of physical behaviour (lines 344 – 350). |
| • Why hasn’t the regression analysis been conducted also for sedentary behaviour and LPA? Please explain the reasons in the text. | We have added to the results section the outcome of performing regression that includes all physical activity (LPA+MVPA) or states 2-5. (Lines 278 – 281). We have not included LPA on its own (LPA on its own is difficult to interpret in the context of the overall abilities of a child – for example a child could have below average participation in LPA because they are very sedentary or because they spend more to in MVPA), the reasoning is the same for HSMM states 2-3. Sedentary behaviour is equivalent to (1 – total activity) and so we have not included it. Discussion of this is provided at lines 336 – 343. Because we are using the PEDI-CAT (a measure of disability which is likely to relate more strongly to participation in more vigorous forms of activity) as a comparator, we also considered that time spent in MVPA or intense activity states would be a better predictor of this than sedentary time. |
| Further comments  • Have you considered tuning the parameters of the observations/durations distributions? If not, what is the reasoning? | We used the parameters and distributions recommended by a previous study (Van Kuppevelt, D. et al., 2019) and found that they produced a state-space that we could interpret. We did alter the number of states permitted in the model – reducing this to 6. This was to aid in the interpretation and reduce the computational complexity of the model. Future work should explore the parameters of the distributions but for a dataset of our size the computational resources required to do this would be great. Our model took around 12 hours to run on for a single parameter set on a high-performance computer. Van Kuppevelt, D. et al. (2019) ‘Segmenting accelerometer data from daily life with unsupervised machine learning’, PLoS ONE, 14(1), p. e0208692. doi: 10.1371/journal.pone.0208692. |
| Reviewer #3: The study is interesting. Especially, the problem is well-stated. The manuscript, however, has several errors here and there. The manuscript should be prepared better. There is no conclusion and limitations of the study. The authors should read the manuscript carefully and fix all the grammatical and typos. | Thank you for reading our paper and for your kind comments. We have re-read the manuscript to check for further errors. We have also added limitations to our discussion and we have also included a conclusion. |
| Abstract is written well. Still, the authors should mention what type of accelerometers was used? Where was it worn? Does the accelerometer provide raw acceleration data? | We have updated the abstract to specify the make and model of the accelerometer and how it was worn and to specify that the raw acceleration data was used. Line 28. |
| Line 65. Revise the sentence. Wearer’s is not commonly used, and sound weird. This should be also applied consistently to other sentences using similar terminology. | Updated to say: “Accelerometers are increasingly used to measure physical activity.” Other sentences using the term wearer have also been revised. Line 68. |
| Line 91. I recommend that this sentence should be revised. I think you are not ‘wishing’ but demonstrating | Updated to say demonstrate. Line 97. |
| Line 150. Perhaps a comma is needed after to do this, | Added comma |
| Accelerometer data pre-preprocessing: Can the data clarify how they have made these decision when processing the accelerometer data. Especially, proper references are needed. There is almost no references in this section, making it difficult to understand whether these methodological decisions are justifiable. | Additional comments and references have now been added to this section to indicate sources used for methodological decisions. Line 155 – 181. |
| I was wondering if the authors could provide a figure to visualize how they cleaned the data and used HSMM for this problem. | We have added a supplementary figure (S1) which illustrates part of the data cleaning process – showing the identification of wear time and the selection of consecutive wear days. The application of the HSMM is best illustrated through figure 2 B. |
| Overall, I think the authors should provide references for many of their statements, especially in the methods. e.g Line 183  Line 193. I failed to understand what the authors mean by 10% of an LPA bour and 20% of an MVPA bour to be an interruption? | Updated to read: “This allowed 10% of an LPA bout or SED bout and 20% of an MVPA bout to be outside of the intensity range for that cut point (e.g. less than 28 counts per second for MVPA). By ignoring these interruptions, we are recognising that periods of activity may have short pauses.” Lines 223 – 225. Additional references added throughout methods. |
| Figure 3. Why the authors did not use states 1-3, when doing the regression? This also the case when looking at Table 2. | We have added to the results section the outcome of performing regression that includes all physical activity (LPA+MVPA) or states 2-5. Lines 278 – 281. We have not included LPA on its own (LPA on its own is difficult to interpret in the context of the overall abilities of a child – for example a child could have below average participation in LPA because they are very sedentary or because they spend more time in MVPA), the reasoning is the same for HSMM states 2-3. Sedentary behaviour is equivalent to the inverse of total activity and so we have not included it. Lines 336 – 343. |
| Again, the discussion needs improvement and proper reference. For example, Line 274-275, the authors say in contrast to cut points approaches, but there are no references in the whole paragraph | We have made significant improvements to the discussion, including more thorough referencing throughout. |
| There is no conclusions. Also, limitation and strength should be also added. | We have added a substantial limitations section to the discussion – the strengths are addressed in the general discussion. We have also added a conclusion. |
